# Spontaneous Ovarian Hyperstimulation Syndrome with FSH Receptor Gene Mutation: Two Rare Case Reports

**DOI:** 10.1155/2018/9294650

**Published:** 2018-10-15

**Authors:** Emsal Pinar Topdagi Yilmaz, Omer Erkan Yapca, Yunus Emre Topdagi, Seray Kaya Topdagi, Yakup Kumtepe

**Affiliations:** ^1^Department of Gynecology and Obstetrics, Atatürk University School of Medicine, Erzurum, Turkey; ^2^Clinic of Gynecology and Obstetrics, Nenehatun Gynecology and Obstetrics Hospital, Erzurum, Turkey

## Abstract

Development of ovarian hyperstimulation syndrome (OHSS) is very rare in a spontaneous ovulatory cycle and it is usually seen during pregnancy. In the etiology of OHSS, higher hCG (molar pregnancies or multiple pregnancies) and thyroid-stimulating hormone (TSH) levels have been accused. In recent years, some follicle-stimulating hormone (FSH) receptor (FSHR) gene mutations have been described in patients with OHSS in the first trimester with normal hCG levels. Herein, we report two cases of FSHR gene mutation during the investigation of the etiology of spontaneous OHSS. Although OHSS is typically associated with ovulation induction, it should be kept in mind that this condition may also develop in spontaneous pregnancies.

## 1. Introduction 

Ovarian hyperstimulation syndrome (OHSS) is a serious complication developing after the gonadotropin use for assisted reproduction methods and is observed in 10% of patients [1]. It is more common after the implantation procedure, particularly, and is accompanied by increased miscarriage rates [1–4]. The incidence of iatrogenic OHSS is 0.2 to 1%, and the risk of mortality is 1:45000-1:50000 [5].

When gonadotropin therapy is not used, the development of spontaneous OHSS is quite rare and it is usually associated with pregnancy. In the etiology of OHSS, higher hCG (molar pregnancies or multiple pregnancies) and thyroid-stimulating hormone (TSH) levels have been accused. In recent years, some follicle-stimulating hormone (FSH) receptor (FSHR) gene mutations have been described in patients with OHSS in the first trimester with normal hCG levels [6]. Clinical findings include massive ovarian growth, abdominal distension, nausea and vomiting, abdominal and inguinal pain, loss of protein rich fluid to the third space due to increased capillary permeability, extensive abdominal ascites, dyspnea, electrolyte imbalances, and the risk of thromboembolism and oliguria [3, 4]. In the differential diagnosis of OHSS, conditions such as luteoma of pregnancy, recurrent theca lutein cysts, ovarian cancer, and hyperreactio luteinalis should be considered.

Herein, we report two cases of severe OHSS during spontaneous first trimester pregnancy in whom a FSHR gene mutation has been discovered by the genetic testing. We discuss this rare spontaneous OHSS clinical picture in the light of current data.

The test method used is as follows: the 5, 6, 7 and 10 exons in the FSHR gene and the exon/intron combination are applied. The results were analyzed using the Mutation Surveyor Program.

## 2. Case Report 1

**Figure 1:**
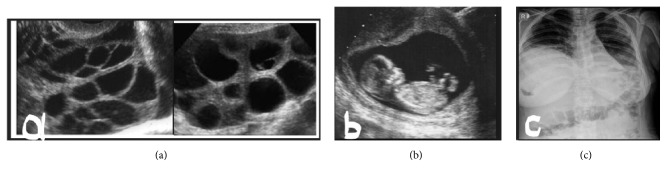
(a) Multicystic appearance in both ovaries (left ovary, 14*∗*13 cm; right ovary, 13*∗*12 cm). (b) A single intrauterine pregnancy with gestational age of 12 weeks according to the crown-rump length. (c) On chest X-ray, the costophrenic angles were closed with appearance of hydrothorax.

A 20-year-old woman (Gravida 2/Para 1) who was unaware of the date of the last menstrual period and was evaluated during routine pregnancy follow-up presented with nonspecific complaints, such as abdominal pain, bloating, dyspepsia, and occasional respiratory distress. The medical and gynecological history of the patient was unremarkable. She had regular menstrual cycles and did not use oral contraceptives or any medication for the ovulation induction. An increase in the bilateral ovarian size (left side 14x13 cm and right side 13x12 cm) and a multicystic appearance were observed (Figure 1(a)). Ultrasonographic evaluation revealed a single live intrauterine fetus of 12 weeks gestation (Figure 1(b)). Extensive fluid was seen in the abdominal cavity. On chest X-ray, the costophrenic angles were closed and an appearance of hydrothorax was observed (Figure 1(c)). On physical examination, there was abdominal distension and tenderness. The laboratory tests of the patient were as follows: quantitative hCG 117740 IU/ml, TSH 3,229 *µ*IU/ml, and free T3 and T4 were within normal ranges; hemoglobin 16,7 g/dl, hematocrit 47,6%, E2 >5000 pg/ml, PT, PTT, and fibrinogen were within normal limits; routine biochemical tests were normal (for example, total protein, albumin, creatinine, BUN, Na, K, AST, ALT, and LDH); interestingly CA-125 (564 IU/mL) was found higher, Inhibin A 861, Ristocetin cofactor (von Willebrand factor (VWF) activity) 100% (50-100%), and VWF antigen 150% (60-150%).

Serological tests (anti-HAV IgM, HBsAg, and anti-HCV) were found to be negative. Antithrombin 3, lupus anticoagulant, protein C and S activity, antiphospholipid antibodies (IgM and IgG), and anticardiolipin antibodies (IgM and IgG) were found to be within normal ranges in thrombophilia screening. Factor V Leiden mutation was not observed. Other causes of spontaneous OHSS were ruled out. In the examination of FSHR gene mutation due to investigation of spontaneous OHSS, a mutation was identified which has been previously described and reported as a disease-related mutation. The result is shown in Table 1. Doppler ultrasonography revealed normal arterial blood flow in bilateral ovaries. The patient was hospitalized with the diagnosis of Grade 2 spontaneous OHSS, according to the Golan classification [7], and conservative treatment was initiated.

Daily 75 mg of rectal indomethacin, 1500 cc/day intravenous saline infusion adjusted by considering the electrolyte balance, follow-up of fluid input and output, daily measurement of weight and waist circumference, and 3500 IU/day of low molecular weight heparin (tinzaparin sodium) for thromboembolism prophylaxis were administered. During the hospital stay, the clinical symptoms of the patient, such as abdominal pain and distention, increased. Repeated abdominal ultrasonography revealed advanced ascites in the abdomen. Approximately 2.5 to 3 L of ascites fluid was drained with a spinal needle by paracentesis. The treatment was supplemented with 20% 50cc albumin solution. Within the next three days, the laboratory parameters and clinical symptoms of the patient improved and she was discharged with suggestions of follow-up antenatal outpatient clinic visits. At 38 weeks of pregnancy, the patient gave birth of a 2,950 g healthy alive male baby, and in the first postpartum month, both ovaries were in normal appearance in transvaginal ultrasonography and in Doppler examination. The extensive abdominal ascites fluid also completely resolved. She had no complications during pregnancy and postpartum period.

## 3. Case Report 2

**Figure 2:**
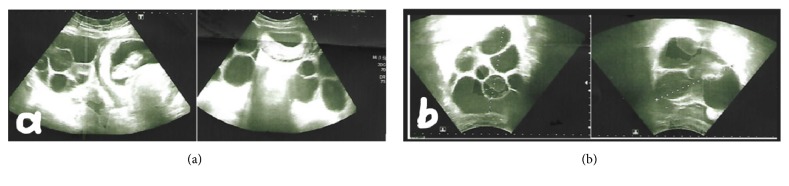
(a) Intrauterine pregnancy with gestational age of 10w and multicystic appearance in left ovary. (b) Bilateral ovaries (left ovary, 12*∗*12, 5cm; right ovary, 11*∗*13cm).

A 28-year-old woman (Gravida 3/Para 1) with unremarkable medical and gynecological history had regular menstrual cycles and did not use oral contraceptives or any medication for the ovulation induction. Ultrasonographic evaluation revealed a single live intrauterine fetus of 10-week gestation (Figure 2(a)). An increase in the bilateral ovarian size (left side 12x12,5 cm and right side 11x13 cm) and a multicystic appearance were observed (Figure 2(b)). The patient's laboratory tests were similar to those of the patient in the first case. Other causes of spontaneous OHSS were ruled out. In the examination of FSHR gene mutation due to investigation of spontaneous OHSS, a mutation was identified which has been previously described and reported as a disease-related mutation. The result is shown in Table 1.

The patient was hospitalized with the diagnosis of Grade 2 spontaneous OHSS, according to the Golan classification, and conservative treatment was initiated. At 40 weeks of pregnancy, the patient gave birth of a 3840 g healthy alive female baby. She had no complications during pregnancy and postpartum period.

## 4. Discussion

Spontaneous OHSS can occur both in pregnant and nonpregnant women. De Leener classified spontaneous OHSS syndrome into three types based on clinical presentation and FSH receptor mutation. Type I is associated with the mutated FSH receptor and this type may cause recurrent spontaneous OHSS. Type II is secondary to high levels of human chorionic gonadotropin (hCG) as in hydatiform mole and multiple gestation and is the most frequent one. Type III is related to hypothyroidism[6].

Mutations in the FSH receptor (FSHR) could be activating, leading to a predisposition to OHSS, or inactivating, resulting in sterility due to poor ovarian response to gonadotropins. Polymorphisms of FSHR have been investigated and about 744 single nucleotide polymorphisms have been identified in the FSHR gene, of which only eight are located in the coding region, exons, with the rest being intronic. Ovarian response is dependent on the FSHR genotype. Clinical studies on the p.N680S polymorphism of the FSHR gene have demonstrated the homozygous Ser/Ser variant to be less sensitive to endogenous or exogenous FSH in terms of estradiol production. Polymorphism of the FSHR, Ser680Asn, in the FSHR gene is a predictor of the severity of symptoms in patients who develop OHSS [8, 9].

FSHR p.Ala307Thr and p.Ser680Asp polymorphisms have been associated with low over-reserve response and have been reported to cause prolongation of the ovulation induction period[10]. When the change in position 680 of the FSHR gene (p.Asn680Ser) is detected homozygously, however, the desired E2 level can be reached by administering a higher dose of exogenous FSH[11]. Recently, the inactivation of FSHB and FSH-R genes in animal models has been shown to have an effect on FSH hormone levels. Mutations in FSHB and FSHR genes have also been observed in a group of patients with similar phenotypic effects[12].

FSHR-activating mutations: c.383C>A(exon 5), c.1345A>G, c.1346C>T, c.1634T>C, c1699G>A, c.1700A>G (exon 10).

FSHR-inactivating mutations: c.479T>C (exon 6), c.566C>T (exon 7), c.1255G>A, c.1555C>A, c.1717C>T, c.1760C>A, c.1801C>G (exon 10).

The knowledge about the pathophysiology of OHSS has been steadily increasing, and many agents have been suggested to play a role in the etiology of OHSS, such as estrogen, histamine, prostaglandins, aldosterone, renin, angiotensin II, and vascular endothelial growth factor (VEGF) [4]. The development of spontaneous OHSS in pregnancy is a rare clinical presentation. In recent publications, three distinct gene mutations in the FSHR have been discovered in patients with recurrent severe spontaneous OHSS. These mutated receptors are highly sensitive to hCG* in vitro* [13–15]. In normal pregnancy, FSHR cannot be stimulated or becomes very weak due to the very low amount of pituitary gonadotropins in the serum. In spontaneous OHSS patients, hyperstimulation develops with pregnancy-induced hCG due to increased and mutated FSHR in the growing follicle [15]. In another study of FSHR mutations, it was reported that the presence of 680FSHR mutation was predictive in determining the clinical severity of OHSS [16]. In our two cases, FSHR gene mutation was examined during the investigation of spontaneous OHSS, and it was positive. Other causes of spontaneous OHSS were ruled out.

The hCG in the blood reaches maximum level at 8th to 10th gestational weeks and starts to fall. In addition, in clinical follow-up, spontaneous OHSS develops after the 8th gestational week. However, iatrogenic OHSS cases are seen at earlier gestational weeks due to exogenous hCG stimulation [16]. In our two cases, similarly, spontaneous OHSS developed approximately at the 10th to 12th gestational week.

In the literature, there are also cases showing the coexistence of spontaneous OHSS and hypothyroidism, and polycystic ovary syndrome [16]. Possible risk factors such as molar pregnancy, multiple gestation, polycystic ovary syndrome, and hypothyroidism to explain the development of spontaneous OHSS were not found in our two cases.

In the literature, there are many studies on pregnancy and management of OHSS. Accordingly, the treatment of OHSS should be conservative and include the regulation of intravascular volume and electrolyte balance and the prevention of complications such as hemoconcentration, hypovolemia, and coagulation disorders [17]. It has been reported that the miscarriage rates increase in pregnancies with the development of these complications. Since OHSS is usually self-limiting, the continuation of pregnancy is recommended. In almost all cases, pregnancy has reached term. Very rarely, deaths due to hypovolemia, hemorrhage, and thromboembolic events have been reported [18].

Terminating the pregnancy may be considered in cases resistant to conservative treatment, and emergency laparotomy may be necessary in conditions such as ovarian rupture, torsion, and intraperitoneal hemorrhage. Briefly, the identification of risky patients, preference for prevention strategies, bed rest, albumin levels with low salt, aspiration of ascites fluid by transvaginal ultrasonography, furosemide, low sodium intake, dopamine, close monitorization, and, recently, dopamine agonists are used in the treatment. In addition, studies are ongoing on the VEGF, which plays a major role in the pathophysiology of OHSS.

Contrary to previous studies on spontaneous OHSS management, in our two cases, the severity of OHSS was moderate and did not cause any pregnancy complication; thus, this finding supports recent publications [18]. In spontaneous OHSS cases, a rare form of OHSS, the diagnosis should be made urgently with clinical findings, unnecessary surgical procedures should be avoided, and conservative treatment for severe and life-threatening complications should be immediately initiated. Spontaneous OHSS cases are relatively more benign than iatrogenic cases. It has been reported that the probability of recurrence in later pregnancies is high. However, it has been reported that spontaneous OHSS may develop even in the patient who had a previous normal pregnancy, as in the presented case [17].

## 5. Conclusion

In conclusion, although OHSS is typically associated with ovulation induction, it should be kept in mind that this condition may also develop in spontaneous pregnancies. As it is usually self-limiting, continuation of the pregnancy is recommended. In general, the treatment recommendation is supportive care and anticoagulant treatment to prevent serious complications, such as pulmonary embolism due to deep venous thrombosis.

Rarity of this condition may also be due to misdiagnosis that can result in mismanagement or severe complications of this condition.

## Figures and Tables

**Table 1:** The result of FSHR gene mutation in the presented two cases with spontaneous OHSS.

Case report 1	c.383 C>A p.S128Y	Homozygous
	c.733 G>C p.Ala307Thr	Heterozygous
	c.1961 G>C ve c.-29 G>A p.Ser680Asp	Polymorphism

Case report 2	c.383 C>A p.S128Y p.Ser128Tyr	Heterozygous
